# Digital health, cardiometabolic disease and ethnicity: an analysis of United Kingdom government policies from 2010 to 2022

**DOI:** 10.1057/s41271-023-00410-z

**Published:** 2023-04-21

**Authors:** Zareen Thorlu-Bangura, Lydia Poole, Harpreet Sood, Nushrat Khan, Fiona Stevenson, Kamlesh Khunti, Paramjit Gill, Madiha Sajid, Wasim Hanif, Neeraj Bhala, Shivali Modha, Kiran Patel, Ann Blandford, Amitava Banerjee, Mel Ramasawmy

**Affiliations:** 1 Institute of Health Informatics, UCL, 222 Euston Road, London, NW1 2DA UK; 2 Department of Psychological Interventions, School of Psychology, University of Surrey, Guildford, Surrey UK; 3 Hurley Group Practice, London, UK; 4 Department of Primary Care and Population Health, University College London, London, UK; 5 Diabetes Research Centre, Leicester General Hospital, University of Leicester, Leicester, UK; 6 Warwick Medical School, University of Warwick, Coventry, UK; 7 Patient and Public Involvement Representative, DISC Study, London, UK; 8 Department of Diabetes, University Hospital Birmingham, Birmingham, UK; 9 Institute of Applied Health Research, Queen Elizabeth Hospital Birmingham, University Hospitals Birmingham NHSFT, Edgbaston, Birmingham, UK; 10 University Hospitals Coventry and Warwickshire, Coventry, UK; 11 UCL Interaction Centre, London, UK

**Keywords:** Cardiometabolic, Health inequalities, Digital health, e-health, m-health, Policy

## Abstract

Recent health policies in the United Kingdom (UK) and internationally have focussed on digitisation of healthcare. We examined UK policies for evidence of government action addressing health inequalities and digital health, using cardiometabolic disease as an exemplar. Using a systematic search methodology, we identified 87 relevant policy documents published between 2010 and 2022. We found increasing emphasis on digital health, including for prevention, diagnosis and management of cardiometabolic disease. Several policies also focused on tackling health inequalities and improving digital access. The COVID-19 pandemic amplified inequalities. No policies addressed ethnic inequalities in digital health for cardiometabolic disease, despite high prevalence in minority ethnic communities. Our findings suggest that creating opportunities for digital inclusion and reduce longer-term health inequalities, will require future policies to focus on: the heterogeneity of ethnic groups; cross-sectoral disadvantages which contribute to disease burden and digital accessibility; and disease-specific interventions which lend themselves to culturally tailored solutions.

**Supplementary Information:**

The online version of this article contains supplementary material available 10.1057/s41271-023-00410-z.

## Key messages

- UK government policies increasingly emphasise the benefits of digital health, including for prevention, diagnosis and management of cardiometabolic disease.
- While the COVID-19 pandemic amplified increasing digitisation and awareness of health inequalities, no policies address ethnic inequalities in digital health for cardiometabolic disease, despite high prevalence in minority ethnic communities.
- Future policies can promote digital inclusion and reduce longer-term health inequalities by taking into account intersecting disadvantages and opportunities for culturally tailored solutions.

## Introduction

Digital health technologies (including mobile-health or m-health, e-health and tele-health) encompass the application and use of electronic or digital resources to aid and facilitate healthcare services and interventions [1]. Examples of digital health include digitisation of patient medical records, wearable electronic devices and online health consultations. The World Health Organization’s (WHO) global strategy on digital health suggests that all countries need to prioritise incorporation of digital technology in their healthcare sectors [2]. The United Kingdom (UK) government has demonstrated its commitment to digital healthcare via publication of a digital inclusion strategy [3], a Digital Inclusion Charter [4], and, more recently, Public Health England’s (PHE) 'digital first’ strategy: ‘integrating digital ways of working into the design of external and internal products, services and business processes’[5]. Such approaches aim to provide equitable access to healthcare by incorporating digital technologies, with the intention of reducing health inequalities [5]. Digital health interventions and strategies for digital inclusion could be particularly useful to tackle cardiovascular disease (CVD) and diabetes mellitus (together constituting “cardiometabolic disease”), which represent two leading causes of disease burden in England [6] and frequently present as comorbidities due to shared risk factors [7]. Prevalence of diabetes and CVD vary by ethnic background [8]. Black and South Asian groups have higher prevalence and increased mortality risk from diabetes and CVD in the UK [9] and such associations have persisted over the past few decades [10]. During the pandemic, population data analyses related to COVID-19 hospitalisation have highlighted the unequal burden of morbidity and mortality faced by minority ethnic groups in the UK [11], prompting targeted policies and interventions.

Benefits of digital healthcare include the provision of information to empower the public to prevent ill health or manage health conditions, and increase access and ease of interaction with public services [12]. However, attempts to promote digital healthcare may overlook pre-existing inequalities in digital access, digital literacy and digital health literacy, particularly among minority ethnic groups in the UK [13]. While there is limited evidence for efficacy of digital health approaches to tackle inequalities, a review (2020) in the UK shows examples focused on providing online access and support for those not engaging with health services, such as those experiencing homelessness, and young people of minority ethnic backgrounds who are overweight or at risk of diabetes [14].

Improved understanding is hampered by a lack of systematic analysis regarding the extent to which digital health policies take into account the following: (a) specific diseases and (b) ethnic health inequalities. Rapid progression of digital health in the UK’s National Health Service(NHS) as a result of the COVID-19 pandemic, coupled with increased awareness of ethnic differences in COVID-19 outcomes, has prompted critical review of failure of existing policies to tackle health inequalities [15]. Therefore, it is opportune to analyse government policies to investigate direct government action aimed at health inequalities, using cardiometabolic disease as an exemplar.

We conducted a content analysis of UK policy documents, introduced since 2010, both pre- and post-COVID-19 pandemic, to review national and regional government initiatives to quantify, understand and address the intersection of ethnicity, cardiometabolic disease and digital health [16]. We described search strategy, information sources and analysis in the Supplementary Material Methods and Appendices 1–4.

## Results

In total, we included 87 policy documents in the qualitative content analysis (see Supplementary Material Appendix 4). Policies we included targeted multiple stakeholders: system-wide aimed at health and social care, or public health (*n* = 40), health data and digital health (*n* = 15), digitisation (*n* = 6), cardiometabolic disease (*n* = 4), COVID-19 (*n* = 4), public health or other health conditions (*n* = 2) and related to inequalities, including health (*n* = 15) (see Appendix 4). Health is a devolved power in the UK; 17 policies specifically stated they were UK-wide (specifically stated); the remainder related to Scotland (*n* = 15), Wales (*n* = 9), Northern Ireland (*n* = 7), local authorities or NHS regions (*n* = 4) and England (*n* = 34).

Analysis of these documents set out the context for existing policies. We considered the extent to which the three areas relating to digital health, ethnic inequalities and cardiometabolic disease are considered together within existing policies. We explored these under the following five themes: policy context for digital health inequalities; representation of ethnicity and ‘marginalised’ groups; addressing digital accessibility, skills and literacy to enable digital health; and the potential for digital in cardiometabolic health conditions. Embedded in each of these themes is the impact of COVID-19, which emphasised ethnic inequalities in health and prompted government action and policy.

### Policy context: digitisation of health is embedded in policy and action.

This theme describes the broader context underpinning the specific policies pertaining to each of our three areas for inclusion (cardiometabolic disease, digitisation and inequalities/minority ethnic groups). During the period covered in this review, we observed a continued focus on these three areas of interest in high-level strategy documents for the whole of the health care system (*n* = 32) (see Supplementary Material Appendix 4, Sect. 1). For example, the first mandate from the Government to NHS England from April 2013 to March 2015 set out expectations and objectives for delivery of health and care in England. This highlighted the need to increase use of technology to help people manage their health and care; the importance of prevention, diagnosis, treatment and management of cardiometabolic disease; and the legal duties of NHS England to tackle health inequalities and advance equality [17]. Similarly, the first objective of the first PHE ‘Our Priorities’ document for 2013/14 was to reduce preventable deaths and the burden of ill health associated with smoking, high blood pressure, obesity, poor diet, poor mental health, insufficient exercise and alcohol; and elsewhere the document referenced the need to tackle the wider determinants of health [18]. The first mandate to Health Education England highlights the role of health and care staff in addressing health inequalities[19]. In subsequent years, government developed these aspects further. In 2017, the NHS mandate included the role of commissioning in tackling health inequalities [20]; and in 2018, both the NHS mandate and PHE remit highlighted the potential of embedding prevention of ill health (including of cardiovascular conditions and obesity) as a way to reduce health inequalities [21].

Against this backdrop, we identified four main ways in which documents described the origins of specific policies: system drivers, such as a move to digital in other national institutions; new and emerging evidence; availability of funding; and public or stakeholder opinions. For example, the creation of the Commission on Race and Ethnic Disparities, supported by the Race Disparity Unit of the Cabinet Office, took place in a context of increased discourse and public concern about ethnic health inequalities during the pandemic, and in the wake of Black Lives Matter protests.

Policymakers have embedded digitisation of health in policy development since 2010, with specific policies introduced across England [22], Wales [23] and Scotland [24]. We identified six strategies related to digitisation of public services highlighting health and care as a key area for change [25–30]. The COVID-19 pandemic has driven creation of policies focused on health digitisation at both regional and national levels [27, 28], and use of the NHS apps to support patient access to data [31], and for patients to organise and manage their own health and care [32]. The NHS has embedded these policies into practice including data linkage between primary and secondary care [33], use of electronic booking systems [34], digital consultations [35] and increased uptake of the NHS app [36]. In addition, COVID-19 has also resulted in planning beyond the pandemic, with optimism surrounding the continued inclusion of digital tools within the NHS: “The pandemic has shown the positive changes we can make to how and where we deliver healthcare, and we must lock that in. By accelerating the transition to a new model of community NHS care and supporting the digitisation of services, such as NHS Near Me, we will ensure people get the right care, in the right place, at the right time” [35].

**Fig. 1:**
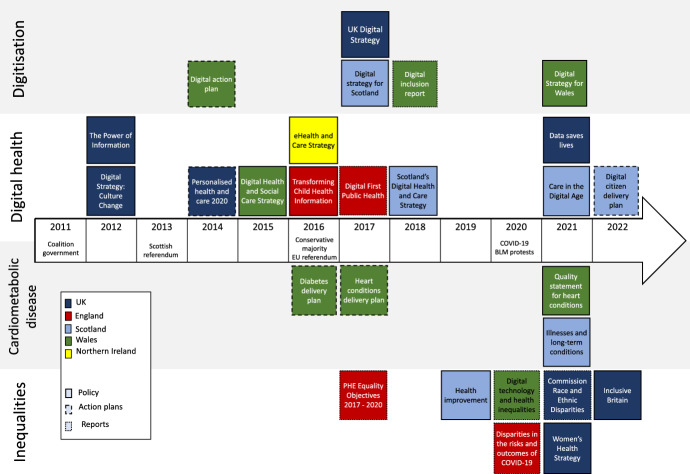
Timeline of the key policies related to cardiometabolic disease, digital health, and inequalities

In subsequent sections, we consider the limited focus on ethnicity within policy documents, and given this context, approaches to addressing digital inequalities to enable digital health, and the intersection between cardiometabolic disease, digital health and ethnicity (see Fig. 1 for a timeline of the key policies related to our areas of interest that were published in this period).

### Representation of ethnicity and ‘marginalised’ groups

This theme focuses on consideration given to specific ethnic groups or communities within policies related to digital health and cardiometabolic disease. We explored this through analysis of language and context, and the degree to which documents included specific and culturally tailored examples of good practice.

Prior to 2020, no documents focussed exclusively on ethnicity and health. Their drafters used the term ‘BAME’ (Black, Asian and Minority Ethnic) in nine of the total documents [14, 37–44], with no or limited focus on specific ethnic groups in the UK. Policy drafts used ‘BAME’ interchangeably and in conjunction with other terminologies such as ‘disadvantaged backgrounds’ [45][46][47–51]; in Northern Ireland, this focuses on regionally relevant characteristics related to inequalities, gender and geographical area, as at the last census, less than 2% of the population identified as belonging to a minority ethnic group [52].

The impact of COVID-19 on minority ethnic communities in the UK prompted shifts in language and a renewed focus on ethnicity. Ethnicity and health were key areas of interest for both the Commission on Race and Ethnic Disparities (which called for the end of the use of the term ‘BAME’ in Government [42–44]) and for the PHE report on Disparities in the Risk and Outcomes of COVID-19 [41]. Actions included publication of the first Advancing Mental Health Equalities Strategy in October 2020, development and testing of a Patient and Carers Race Equality Framework [53], and increasing support for research in “more diverse and under-served populations” [54]. A number of responses to a call for evidence from the Commission on Race and Ethnic Disparities considered racism a cause of ethnic disparities in healthcare, policing, education and employment, and identified actions they would like to see from national and local government [55]. Additionally, a number of COVID-19 policies targeted improving outcomes in minority ethnic groups, including the following: establishing a dedicated team to support effective communication with minority ethnic staff in the NHS [56], creating an Equalities Board for inclusive vaccine delivery based on the principle of inclusion [56] and showcasing local engagement with minority ethnic communities [57]. Given the limited focus on ethnicity within the documents identified in the review, the remaining themes focus on the broader consideration of digital and health inequalities.

#### Addressing digital accessibility, skills and literacy to enable digital health

This theme focuses on digital inequalities and captures the extent to which the policies show consideration for the digital requirements (in terms of both the physical infrastructure and the individual) to support the roll out of any new initiatives. This sets out a broader digital context in which digital health approaches in cardiometabolic disease are proposed and implemented. We identified content regarding the quality of the available general digital infrastructures, access to digital resources (in health and other areas), barriers to access as well as any examples of the types of digital resources currently available.

The Department of Health and Social Care (DHSC) Information Strategy in 2012 set out actions to achieve improvements in the collection and use of information in the health and care system, noting that it must be ‘digital first’ (rather than ‘digital by default’), meaning that while digital methods should be adopted to deliver healthcare, face-to-face contact with health and care professionals are an essential part of care [22]. In the same year, they announced their ambition to become a ‘Digital First’ department, leading a culture change in the health and care sector [58]. More recent government publications, such as the PHE ‘digital first’ strategy, do not define this term [5].

Several policies demonstrated awareness of the need to improve digital access [14, 28, 30, 32, 59]. For example, the NHS (England) Five Year Forward View published in 2014 identified a need to develop “partnerships with the voluntary sector and industry to support digital inclusion” [32]. In Wales, the digital strategy explicitly mentioned digital inclusion; the document recognised lack of internet affordability as a barrier to inclusion [30]. Beyond cost, it identified a need to “understand the links between digital exclusion, data poverty and financial and social exclusion” [30]. Recognising that some of the population cannot use digital services because they lack the skills and competence, the Welsh government ran a digital inclusion programme, ‘Digital Communities Wales’, from 2015 to 2019, focusing on the most deprived areas. The strategy explicitly stated an intention to “ensure no one is left behind as we embrace a digital first approach, keeping digital inclusion at the heart of all we do” [30]. The COVID-19 pandemic has also accelerated the need to increase digital access to ensure equitable service provision across society [30, 35].

Some policies aimed at improving digital access have also focussed on professional groups and settings in health and social care [35, 60]. Leeds City Council specified the importance of digital access for healthcare professionals; the Leeds Care Record (an electronic resource to improve access to patient records for professionals) that operates across health and care services aims to provide the most comprehensive and up-to-date information about patients to clinicians, thereby reducing the number of times patients are required to divulge sensitive information [60].

#### The potential for digital in cardiometabolic health conditions

This theme focuses on policies that address cardiometabolic disease in the digital space. Four policies explicitly targeted cardiometabolic disease, including specific delivery plans for diabetes [61] and heart conditions in Wales [62, 63], and policy actions for diabetes, heart disease and stroke in Scotland [64]. England set out ambitions for care for cardiovascular disease, stroke and diabetes in the NHS (England) Long-Term Plan [65] and other broader documents. For example, in 2014, NHS England’s ‘Five Year Forward View’ suggested that remote monitoring of patients with congestive heart failure with biosensor technology could improve outcomes and reduce hospitalisations [32]. Similarly, ‘Advancing our health: Prevention in the 2020s’ describes plans to utilise new digital resources to improve the cardiometabolic risk of patients [66].

Additionally, eleven documents that looked at the wider benefits of digitalisation in health highlighted cardiometabolic disease as a clinical area in which digitisation could improve patient experiences [23, 27, 29, 33, 35, 36, 58, 59, 67–69]. In 2017, PHE’s digital strategy showcased the digitisation of the Diabetes Prevention Service Programme as an example of how a tailored digital resource had reduced visits to a healthcare provider by up to 25%. In it, theChange4Life Sugar Smart mobile app uses the camera on a mobile phone to scan barcodes on food and visualise added sugar content, and One You addresses health risks in mid-life [5]. NHS Lanarkshire’s digital strategy highlighted hypertension, obesity and diabetes as common conditions for which patients could be supported through Technology-Enabled Care (TEC), with overall aims for TEC to reduce the need for hospitalisation, improve the quality of life, reduce instances of relapse and help to reverse the condition [67]. This aligns with the wider strategy for Scotland where digital improvements included an electronic monitoring and early warning system for cardiac arrests, a clinical decision support system for diabetes [27], a diabetes management patient portal ‘My Diabetes My Way’ [70] and scaling up of remote blood pressure monitoring [35].

The association between cardiometabolic diseases and broader health inequalities was well understood. For example ‘‘… the population is also suffering from the burden of chronic preventable conditions, like obesity, diabetes and heart disease, which are often concentrated in the poorest sections of society” [41], female-specific risk factors [71], and increased risk of some minority ethnic communities of developing type 2 diabetes [66]. Cardiometabolic disease-specific policies also highlighted the need to address these inequalities in service provision, for example in the Diabetes Delivery Plan for Wales states: “Diabetes prevalence is higher in areas of greatest deprivation, and amongst minority ethnic communities. Services should be designed to reduce this health inequality” [61].

The Diabetes Delivery Plan in Wales also highlighted the use of integrated electronic health record data as a route by which prevention programmes can be “targeted at more deprived areas” [61]. However, the Plan presented limited evidence of addressing health inequalities in the design or implementation of these digital opportunities: a case study presented within a scoping review commissioned by Public Health Wales on digital technology and health inequalities demonstrated how health exclusion in diabetes management could be addressed through co-design of a website to support young people self-managing diabetes [14].

## Discussion

### Findings in context

Our policy content analysis sought to examine how UK Government policies since 2010 have integrated digital health, health inequalities (focussing on ethnicity) and cardiometabolic disease. We identified five overarching themes from the documents, namely: policy context and the embedding of digitisation of healthcare in policy and actions; representation of ethnicity and marginalised groups; addressing digital accessibility, skills and literacy to enable digital health; and the potential for digital for cardiometabolic health conditions. Our review sets out, for the first time, the intentions and actions of the UK government with regard to addressing ethnic inequalities in digital healthcare for cardiometabolic disease and considers both the pre-COVID-19 pandemic and peri-COVID-19 response as documented through policy.

The policies included in this review covered England and the three devolved parliaments (Wales, Scotland, Northern Ireland) and included multiple types of documents, including policy or strategy, action plans, mandates to health organisations in the UK (such as the NHS and PHE) and reports. These provide both the context and specific intentions of government policy around digital health, inequalities and cardiometabolic disease across the UK. The policies included in this review emerged in varying contexts. However, they included the following: broad societal or system level influences such as digital innovation within the NHS; new and emerging evidence highlighted in commissioned reports and consultations; public and stakeholder opinions; and funding and investment creating opportunities for new digital health responses. Across the nations, governments have shown a commitment to increasing digital healthcare to address both a perceived need within the healthcare sector and by patients, and to keep abreast of the changing context of healthcare provision during the COVID-19 pandemic. The appetite for change by the government is not surprising given the growth in the digital health sector [72] and earlier commitments to implement change in the use of digital technologies for patient benefit, as evidenced through the commissioning of the Topol review in 2017 [73].

The plans for actions within the policy documents reviewed included both concrete examples of successful interventions that had already been implemented (Change4Life Sugar smart app, electronic care records in Northern Ireland [33, 69]) as well as development of digital provision within healthcare services (Digital First [58]). There has been sizeable financial investment to enact digital transformation policies within the NHS, and a new unit was established to specifically lead on this portfolio (NHSX). Despite this, a National Audit Office report noted that ambitions for a paperless NHS are yet to be fully achieved; the previous national digitisation programme closed without achieving its objectives; and local challenges included outdated IT systems [74]. Researchers have also identified a gap between policy intentions and outcomes in digital health elsewhere through content analyses of other national policies including Iran [16].

### Impact of COVID-19

The digital response to COVID-19 has also featured in some of the more recent policies, signalling a commitment to providing a rapid response to public health threats through harnessing existing technologies and digitisation of previously exclusively face-to-face services [34, 58]. While UK and devolved governments have consistently published on digitisation and digital health, the necessity of growth of digital health during COVID-19 saw new digital health policies published in 2021 from the UK [36], Scotland [75] and a new digital strategy for Wales [30]. Policies cited a range of evidence from across the UK in favour of digital healthcare, with examples from technology-based solutions as well as from the aggregation of big data, but not precision-health approaches that take individual level variation into account [76]. Within the policies, challenges to implementation were less explored, despite recognition from other reports regarding the significant difficulties in integrating new digital healthcare solutions with existing health information technology systems [76].

### Barriers to health digitisation

The policies under review considered barriers such as those surrounding digital accessibility, as well as digital skills and literacy. Earlier reviews have highlighted a lack of understanding, or acknowledgement, of the potential for digital solutions to perpetuate or exacerbate existing health inequalities [12]. It is therefore encouraging that many of the documents showed consideration not only for the physical digital infrastructure required for the increased provision of digital healthcare, but also requirements of the individual user (both healthcare professionals [19, 35, 60] and patients [30]). The Welsh government, in particular, has signalled a commitment to digital inclusion [28] and the need to address area-level deprivation to circumvent financial barriers to digital access and to reduce the so-called digital-divide [77], as well as commissioning a report on digital technology and health inequalities [14]. While the Welsh strategy recognised digital literacy as well as access, it did not mention either health literacy or digital health literacy [30], both of which are important to solution-focused models of digital healthcare implementation [78]. Within the policies in included in the review, discussions of barriers to health digitisation within policies were largely atheoretical, despite strong conceptual models from the scientific community regarding the challenges to scale-up, spread and sustain health technologies [79], pointing towards the complex micro- and macro- level systems in operation.

Our interest in exploring disease-specific policies led us to focus on documents pertaining to digital healthcare for the cardiometabolic diseases. Healthcare technologies are established in this field, due to the population burden of cardiometabolic disease in the UK [80, 81] and the long-term management of both diabetes and CVD [82]. Evident government commitment to invest in cardiometabolic-related technology in the policies we reviewed signalled a broad strategy to ensure sustained advancements for patient benefit [27, 67, 70] and cost-effectiveness [69].

### Consideration of ethnicity

Some documents used the term ‘BAME’ and predate calls for this nomenclature to be dropped [43, 83]. In the policy documents reviewed, the government demonstrates an awareness of ethnic inequalities in healthcare, but there is a lack of granularity to refine ‘minority groups’ to create more targeted approaches. This is particularly important in the cardiometabolic context as there are marked differences in prevalence and outcomes among various South Asian and Black communities. Figure 1 shows how during and post the COVID-19 pandemic, there has been a new focus on health, racial and ethnic disparities. For example, the 2021 Commission on Race and Ethnic Disparities Report [43] and the associated government response, Inclusive Britain [44], describe the need to identify and tackle health disparities by ethnicity and the need to recognise heterogeneity within ethnic groupings such as ‘South Asian’ and ‘Black’. These reports also recognise the need to pay attention to overlapping inequalities according to deprivation and geography, which compound ethnic health inequalities; a white paper for England expected in 2022 to address these issues has since been shelved [84]. Only one document included an example which focussed on a specific minority ethnic group—a case study on the COVID-19 vaccine uptake plan targeting South Asian communities [57]. This is not limited to the UK; others also identified a lack of targeting of population groups most in need in policy analyses for physical activity promotion in European countries [85] and cardiometabolic disease risk factors in South Asian labour migrants in the Middle East [86]. A recent review published by the NHS Race and Health Observatory [87] highlighted disparities between ethnic groups for digital health access. The review included evidence from health practitioners who showed concern over increased digitalisation during the pandemic and the potential to widen health inequalities [87]. None of the documents included explicitly mentioned or brought together ethnic health inequalities in cardiometabolic disease, nor in digital health to prevent and manage cardiometabolic disease, despite the excess disease burden in this group [88].

## Strengths and limitations

In this first content analysis of UK digital health policies, we demonstrate that UK digital health policies are often broad, with limited focus on, or integration with, high-burden diseases such as cardiometabolic disease, and lack specific action on the intersection of these with health inequalities. Our review has several strengths including the use of updated searches, use of multiple perspectives of within research team and application of a rigorous thematic analysis approach. However, we must also acknowledge some limitations. The search strategy resulted in the identification of 87 policy documents from across the UK. Despite using a combination of methods to identify the policies, it is possible we did not capture all, particularly if they were not labelled as a policy on the host website, nor published online. We based our analysis on content provided within the documents and did not supplement it with additional sources, such as reports on policy implementation or impact assessments. This review focussed on the content of existing policies. Future research should also examine policy implementation and translation at national, local and international levels. While most of the policies we identified in this analysis were broad, the focus on cardiometabolic diseases did not allow us to capture divergent content arising in relation to other chronic physical and mental illnesses.

## Conclusion

All four UK nations intend to expand digitisation of the healthcare sector and have policies in place to increase digital access more broadly. However, we found no specific policies addressing ethnic inequalities in digital health for cardiometabolic disease prevention or management. Our findings suggest that to create opportunities for digital inclusion and a reduction in health inequalities, future policies should consider the following: 1) ethnic groups as heterogeneous communities with diverse needs and patterns of disadvantage; 2) overlapping disadvantages which contribute to both disease burden and digital accessibility; and 3) disease-specific opportunities in digital healthcare that lend themselves to culturally tailored solutions.

## Supplementary Information

Below is the link to the electronic supplementary material.

Electronic supplementary material 1 (DOCX 111 kb)

## Data Availability

No new data were generated or analysed in support of this research. The full list of included documents can be found in the Supplementary Material Appendix 4.
