# Correction to: Digital health, cardiometabolic disease and ethnicity: an analysis of United Kingdom government policies from 2010 to 2022

**DOI:** 10.1057/s41271-023-00415-8

**Published:** 2023-05-10

**Authors:** Zareen Thorlu-Bangura, Lydia Poole, Harpreet Sood, Nushrat Khan, Fiona Stevenson, Kamlesh Khunti, Paramjit Gill, Madiha Sajid, Wasim Hanif, Neeraj Bhala, Shivali Modha, Kiran Patel, Ann Blandford, Amitava Banerjee, Mel Ramasawmy

**Affiliations:** 1 Institute of Health Informatics, UCL, 222 Euston Road, London, NW1 2DA UK; 2 Department of Psychological Interventions, School of Psychology, University of Surrey, Guildford, Surrey UK; 3 Hurley Group Practice, London, UK; 4 Department of Primary Care and Population Health, University College London, London, UK; 5 Diabetes Research Centre, Leicester General Hospital, University of Leicester, Leicester, UK; 6 Warwick Medical School, University of Warwick, Coventry, UK; 7 Patient and Public Involvement Representative, DISC Study, London, UK; 8 Department of Diabetes, University Hospital Birmingham, Birmingham, UK; 9 Institute of Applied Health Research, Queen Elizabeth Hospital Birmingham, University Hospitals Birmingham NHSFT, Edgbaston, Birmingham, UK; 10 University Hospitals Coventry and Warwickshire, Coventry, UK; 11 UCL Interaction Centre, London, UK

**Correction to: Journal of Public Health Policy (2023)** 10.1057/s41271-023-00410-z

A piece of funding information was missing from this article and should have read ‘ZTB and A Banerjee were supported by a BMA Foundation TP Gunton grant.’

The original article has been corrected.

