# Supplementary material for: Digital health, cardiometabolic disease and ethnicity: an analysis of United Kingdom government policies from 2010 to 2022
Source: J Public Health Policy. 2023 Apr 21;44(2):179–95. doi: 10.1057/s41271-023-00410-z (PMC10120476; doi:10.1057/s41271-023-00410-z)
Supplement: Supplementary file 1 — Electronic supplementary material 1 (DOCX 111 kb) [file 41271_2023_410_MOESM1_ESM.docx]

**Digital health, cardiometabolic disease and ethnicity: an analysis of United Kingdom government policies from 2010 to 2022**

Zareen Thorlu-Bangura^1^, Lydia Poole^2^, Harpreet Sood^3^, Nushrat Khan^1^, Fiona Stevenson^4^, Kamlesh Khunti^5^, Paramjit Gill^6^, Madiha Sajid^7^, Wasim Hanif^8^, Neeraj Bhala^9^, Shivali Modha^7^, Kiran Patel^6,10^, Ann Blandford^11^, Amitava Banerjee^1*^, Mel Ramasawmy^1*^

1. Institute of Health Informatics, London, UK
2. Department of Psychological Interventions, School of Psychology, University of Surrey, Guildford, Surrey, UK
3. Hurley Group Practice , London , UK
4. Department of Primary Care and Population Health, University College London, London, UK
5. Diabetes Research Centre, Leicester General Hospital, University of Leicester, Leicester, UK
6. Warwick Medical School, University of Warwick, Coventry, UK
7. Patient and Public Involvement Representative, DISC Study, UK
8. Department of Diabetes, University Hospital Birmingham, Birmingham, UK
9. Institute of Applied Health Research, Queen Elizabeth Hospital Birmingham, University Hospitals Birmingham NHSFT, Edgbaston, Birmingham, UK
10. University Hospitals Coventry and Warwickshire, Coventry, UK
11. UCL Interaction Centre, London, UK

**Supplementary Material**

| List of abbreviations | p2 |
| --- | --- |
| Methods   - Search strategy and selection criteria - Information sources - Search - Study Selection - Analysis | p3 |
| Appendix 1. Inclusion and Exclusion Criteria | p5 |
| Appendix 2. Information sources used | p6 |
| Appendix 3. Search terms strategy as used for .gov website | p7 |
| Appendix 4. Full list of included documents | p8 |
| References included in supplementary material | p16 |

**List of abbreviations**

DH – Department of Health (now Department of Health and Social Care, DHSC)

DH (NI) – Department of Health Northern Ireland

DHSC – Department of Health and Social Care

NHS – National Health Service

NHS England – (National Health Service) England, officially the NHS Commissioning Board. Is now merged with NHS Improvement.

NHS Digital – (National Health Service) Digital

PHE – Public Health England, functions now transferred to the UK Health Security Agency (UKHSA) and the Office for Health Improvement and Disparities (OHIC, part of DHSC), NHS England and NHS Digital.

**Methods**

Search strategy and selection criteria

We conducted a systematic search of policies where the main inclusion criteria were regional or national (both UK-wide and individual nation) policy documents published between January 2010 and April 2022. We chose the start date to capture any documents published ahead of the first government digital strategy in health in 2012 [1,2]. We included those documents labelled as a policy document or that appeared under the policy tag on the source website. Due to heterogeneity in types of documents labelled as “policy", we included documents that we determined to meet a definition as ‘policy and strategy’, ‘action plans’ and ‘reports’ [3]. Each document had to reference *at least one* of the following key words (or related constructs): diabetes, cardiovascular, digital health, ethnicity, or inequality. For full inclusion and exclusion criteria, refer to Supplementary Material Appendix 1.

Information sources

One of us (ZTB) systematically searched the websites of the UK national governments and local authorities, NHS and other health-related organisations, and disease-specific health charities to identify relevant documents using embedded search tools, including Google’s site-specific and domain-specific searches. Local and regional government policies appeared in the results as the site-specific search conducted using all 'gov.uk' websites included local council websites. A full list of sites searched is set out in Supplementary Material Appendix 2.

Search

ZTB conducted an initial search in September-October 2020 using the following key terms and related constructs (see Supplementary Material Appendix 3): ‘ethnic*’, ‘digital health’, ‘cardiovascular’ and ‘diabetes’. We used a snowball approach to identify further relevant documents. We repeated these searches in February and April 2022 to ensure capture of new policies which may have been published in response to the COVID-19 pandemic.

Study Selection

Two members of the research team (ZTB and MR) screened all the documents according to inclusion criteria (Supplementary Material Appendix 1). We first screen documents by manual selection based on the title and summary. If we found no summary for the document, we conducted a key word search using the terms: 'digital health' or 'diabetes' or 'cardiovascular disease' or 'minority groups' (or related constructs, as shown in Supplementary Material Appendix 3) to determine relevance of the document. If we deemed a document to be relevant, based on inclusion criteria, we selected it for full-text review. Another author (HS) also reviewed the list of results from the document search to provide suggestions on any key missing documents.

Analysis

The data analysis team included a health sciences researcher (ZTB), a health psychology researcher (LP) and an anthropologist (MR), with clinical oversight (AB), which ensured a transdisciplinary approach to the analysis. ZTB led qualitative content analysis [4] , using an analytic process informed by thematic analysis [5]. We first read documents and analysed them inductively, separately, to enable us to build an understanding of the dataset [5,6]. Familiarisation of the data took place through reading and re-reading of documents. ZTB, LP and MR developed the codes which were then grouped to construct themes and refined through team discussions and testing. We coded transcripts using NVivo 20 (QSR International Pty Ltd.). After conducting initial analysis across the entire dataset, we tabulated themes and subthemes using Excel (Microsoft for Windows 365). Indexing and charting of data ensured rigor in the analysis process. We refined themes with feedback from the broader research team.

**Appendix 1. Inclusion and Exclusion Criteria**

| **Inclusion Criteria** | **Exclusion Criteria** |
| --- | --- |
| Policies published since 2010 | Policies published before 2010 |
| Documents categorised or labelled as policy on the websites | Documents not labelled as policy, e.g. news items and announcements |
| Policy documents published in the UK, England, Scotland, Wales and Northern Ireland | Policy documents published outside the UK |
| Policies referencing digitisation or digitalisation and health | Policies with no reference to digitisation/digitalisation (or related constructs), cardiometabolic illness (or related constructs), inequalities (or related constructs) or minorities (or related constructs); policies which did not refer to health |
| Policies referencing cardiometabolic illness (or related constructs) |  |
| Policies referencing inequalities and health (or related constructs) |  |
| Policies referencing minorities and health (or related constructs) |  |

**Appendix 2. Information sources used**

| UK Government | - <https://www.gov.uk/government/organisations/department-of-health-and-social-care> (*Note that searches raise returns from across UK Government Departments*) - <https://www.england.nhs.uk/> - <https://improvement.nhs.uk/> (*Note this has now been dissolved and incorporated into NHS England*) - <https://www.cqc.org.uk/> - <https://www.nice.org.uk/> - <https://digital.nhs.uk/>   *Google domain-specific search used*   - :nhs.uk (*this also captures other NHS structures such as NHS X, now NHS England Transformation Directorate)* - :gov.uk *(this captures all national and local government pages)* |
| --- | --- |
| Devolved nations of the United Kingdom | *Scotland*   - <https://www.nhsinform.scot/> - <https://www.healthscotland.scot/> - <https://www.nss.nhs.scot/> - <https://www.scot.nhs.uk/> - <https://www.nhslothian.scot/> - <https://tec.scot/>   *Wales*   - <https://www.wales.nhs.uk/> - <https://phw.nhs.wales/>   *Northern Ireland*   - <https://www.nidirect.gov.uk/> - <https://www.health-ni.gov.uk/> - https://online.hscni.net/   *Google domain-specific search used*   - :gov.scot - :gov.wales |
| Disease-specific charity websites | - <https://www.diabetes.org.uk/> - <https://www.bhf.org.uk/> |

**Appendix 3. Search terms strategy as used for .gov website**

| Terms searched |
| --- |
| "Digital health” or “Digital” or “Ehealth” or “MHealth” or “Remote” or “Telehealth” or “Digital health intervention” or “App”  “Diabetes” or “Diabetes Mellitus” or “DM” or “insulin”  Cardiometabolic  “CVD” or “Cardi*” or “Heart disease” or “cardiovascular” or “hypertension” or “blood pressure”  “BAME” or “BME” or “Minority” or “Race” or “Ethnic*” or “Black” or “Asian” or “inequalit*” |

**Appendix 4. Full list of included documents**

| **Ref** | **Title** | **Date of publication** | **Department** | **Region** | **Type** | **Subject** | **Purpose of document** |  |
| --- | --- | --- | --- | --- | --- | --- | --- | --- |
| System-wide policy, strategy or action plans (general) | | | | | | | | |
|  | NHS England mandate   - The Mandate: A mandate from the Government to the NHS Commissioning Board: April 2013 to March 2015 (first mandate) [7] - The Mandate: A mandate from the Government to NHS England: April 2015 to March 2016 [8] - NHS mandate 2016 to 2017 [9] - NHS mandate 2017 to 2018 [10] - NHS mandate 2018 to 2019 [11] - NHS accountability framework 2019 to 2020 [12] - NHS mandate 2020 to 2021 [13] - NHS mandate 2021 to 2022 [14] - NHS mandate 2022 to 2023 [15] | Annual, first published Nov 2012 | Department of Health, subsequently Department of Health and Social care | England | Letter or corporate report | Health inequalities; cardiometabolic disease; digital health | The mandate to NHS England sets out the government’s objectives and budget for the public body. |  |
|  | PHE priorities in health and social care   - Our priorities for 2013/14 [16] - PHE remit letter: 2014 to 2015 [17] - PHE remit letter: 2015 to 2016 [18] - PHE remit letter: 2016 – 2017 [19] - PHE remit letter: 2017-2018 [20] - PHE priorities in health and care: 2018 to 2019 [21] - PHE priorities in health and social care: 2019 to 2020 [22] - PHE priorities in health and social care: 2020 to 2021[23] | Annual | Department of Health and Social care / Public Health England | England | Letter or corporate report | Health inequalities; cardiometabolic disease; digital health | These documents set out the annual priorities for public health in England; these are either in the form of reports from PHE, or letters from the Secretary of State. |  |
|  | The Department of Health and Social Care mandate to Health Education England:   - April 2013 to March 2015 [24] - April 2014 to March 2015 [25] - April 2015 to March 2016 [26] - April 2016 to March 2017 [27] - April 2017 to March 2018 [28] - April 2018 to March 2019 [29] - April 2019 to March 2020 [30] - April 2020 to march 2021 [31] - April 2021 to March 2022 [32] | Annual | Department of Health and Social care | England | Letter or corporate report | Health inequalities; cardiometabolic disease; digital health | Mandate from DHSC sets out strategic objectives for workforce, health education, training and development. |  |
|  | NHS Scotland Chief Executive's Annual Report:   - 2012/2013 [33] - 2013/2014 [34] - 2014/2015 [35] - 2015/2016 [36] - 2016/2017 [37] - 2017/2018 [38] | Annual report | NHS Scotland | Scotland | Corporate report | Digital health; health inequalities; cardiometabolic disease | Annual report on the NHS in Scotland. |  |
| [39] | NHS Five Year Forward View 2014 | 08/03/14 | NHS England | England | Policy paper | Digital health; health inequalities; | Sets out vision for change for the NHS in England and how they attend to achieve it. |  |
| [40] | Our Strategic Plan (Integrated Medium Term Plan) 2019-2022 | Apr-19 | Public health Wales | Wales | Action plan | Health inequalities; cardiometabolic disease | Sets outs short-term steps to implement the long-term strategy for public health in Wales. |  |
| [41] | Advancing our health: prevention in the 2020s | 22/07/2019 | Department of Health and Social Care | England | Consultation paper | Health inequalities; cardiometabolic disease; digital | Consultation on proposals to tackle the causes of preventable ill health in England. |  |
| [42] | PHE Strategy 2020 to 2025 | 10/09/19 | Public Health England | England | Policy paper | Health inequalities; cardiometabolic disease | Sets out public health strategy for England, including priorities for 2020-2025 and how these will be approached. |  |
| [43] | Integration and innovation: working together to improve health and social care for all | 11/02/21 | Department of Health and Social care | UK | Policy paper | Digital health (data); health inequalities | Legislative proposals for Health and Care Bill. Amongst others, includes proposal to make changes to data sharing. |  |
| [44] | Transforming the public health system: reforming the public health system for the challenges of our times | 29/03/2021 | Department of Health and Social care | UK | Policy paper | Public health | Sets out the aims and remits of the new public health agencies in England/UK. |  |
| [45] | Chief Medical Officer for Scotland: Annual Report  2020-21 | Annual report | Scottish Government | Scotland | Corporate report | Digital health; health inequalities; cardiometabolic disease | Annual report on the health of the nation in Scotland. |  |
| [46] | Build back better: Our plan for health and social care | 28/02/2022 | Department of health and social care | UK | Policy paper | Public health | Sets out the introduction of the Health and Social Care Levy, and plans for health and social care post-pandemic. |  |
| Policy, strategies, action plans related to digitisation | | | | | | | | |
| [47] | Digital Action Plan 2014 - 2017 | 20/04/2014 | Welsh Government | Wales | Action plan | Digital (general) | Internal action plan setting out vision for digital, including skills, policy-marking, services and data. |  |
| [48] | UK Digital Strategy | 01/03/2017 | Department for Digital, Culture, Media and Sport | UK | Policy paper | Digital (general) | Digital strategy across all UK economic sectors, includes actions within health and care. |  |
| [49] | Realising Scotland's full potential in a digital world: a digital strategy for Scotland | 22/03/2017 | Scottish government | Scotland | Policy paper | Digital (general) | Update to 2011 Digital Strategy; this focuses on how digital can be embedded across Scotland, including in public services. |  |
| [50] | Improving people’s lives through digital technologies: Digital Inclusion Progress Report and Forward Look 2018 | 2018 | Welsh Government | Wales | Progress report | Digital (inclusivity) | Overview of actions carried out since publication of Digital Inclusion Strategic Framework and Delivery Plan (March 2016) |  |
| [51] | West Midlands Local Industrial Strategy | 16/03/2019 | Department for Business, Energy & industrial strategy | West Midlands | Policy paper | Digital (general) | Sets out strategy for industry in region, highlighting health technology and health data as areas for potential growth. |  |
| [52] | Digital strategy for Wales | 23/03/21 | Welsh Government | Wales | Policy paper | Digital (general) | Sets vision for how digital, data and technology will be used to improve lives. |  |
| Policy, strategies, action plans related to health data and digital health | | | | | | | | |
|  | TEC At a Glance   - 2019 [53] - 2020/21 [54] | 2020 | TEC | Scotland | Corporate report | Digital (health) | Summary of TEC Scotland’s activities in previous year(s) |  |
| [1] | The power of information | 21/03/2012 | Department of Health and Social care | UK | Policy paper | Digital health | Strategy for transformation of information in health and care, including use of electronic care records, and using tech to improve accessibility of services. |  |
| [2] | Digital Strategy: Leading the culture change in health and care | Dec 12 | Department of Health and Social Care | UK | Policy paper | Digital (government) | Ambition to become ‘Digital First’ department; separate from Information strategy for health and care system published in May 2012 |  |
| [55] | Personalised health and care 2020: Using Data and Technology to Transform Outcomes for Patients and Citizens. A Framework for Action | 13/11/14 | National Information Board, Department of Health and Social care | UK | Action plan | Digital health | Describes priorities for the use of data and technology in health and care, and how change will be achieved. |  |
| [56] | Informed Health and Care: A Digital Health and Social Care Strategy for Wales | 2015 | Welsh Government; NHS Wales; ADSS Cymru | Wales | Policy paper | Digital health | Strategy for use of technology for health and wellbeing in Wales, sets out ambitions and priorities for action. |  |
| [57] | eHealth and Care Strategy for Northern Ireland | 02/03/16 | Department of Health | Northern Ireland | Policy paper | Digital health | Five-year strategy for eHealth change, setting out objectives and deliverables. |  |
| [58] | Healthy Children: Transforming Child Health Information | 11/11/16 | NHS England | England | Policy paper | Digital health (data) | Strategy for changes to child health information services, to support parents and professionals in direct care, and promote health and wellbeing. |  |
| [59] | Digital-first public health | 02/02/2017 | Public Health England | England | Corporate report | Digital (public health) | Information on PHE digital strategy for 2017 and 2018. |  |
| [60] | Scotland’s digital health and care strategy: enabling, connecting and empowering | 25/04/2018 | Scottish government | Scotland | Policy paper | Digital (health) | Strategy for digital transformation of health and care, with focus on: planning and improvement of services; enable research and economic development; improved outcome. |  |
| [61] | Digital Strategy 2019-2026 | Apr-19 | NHS Lanarkshire | Lanarkshire (Scotland) | Policy paper | Digital (health) | Overview of strategy for development and implementation of digital services in healthcare |  |
| [62] | Data saves lives: reshaping health and social care with data (draft) | 23/06/21, updated 10/02/22 | Department of Health and Social care | UK | Policy paper | Digital (data) | Strategy for ongoing digital transformation, focusing on commitments for increased role of data in health and care. |  |
| [63] | Enabling, Connecting and Empowering: Care in the Digital Age | 27/10/2021 | Scottish government | Scotland | Policy paper | Digital (health) | Update to 2018 digital health and care strategy, taking into account learning from the pandemic, particularly around digital inclusion. |  |
| [64] | Digital Citizen Delivery Plan 2021/2022 | 2021 | Digital Health and Care Scotland | Scotland | Action plan | Digital (health); Health inequalities | Delivery plan which sets out the strategic priorities for addressing national objectives – addressing inequalities, engaging the population, improving access and promoting wellbeing, and innovation. |  |
| [65] | G7 patient access to health records: final report | 30/12/2021 | Department of health and Social care | UK | Policy paper | Digital (health) | G7 commitment on patient rights to have access to their health information |  |
| Policy, strategies, action plans related to cardiometabolic disease | | | | | | | | |
| [66] | Diabetes Delivery Plan for Wales 2016-2020 | Dec 16 | NHS Wales/Welsh Government | Wales | Action plan | Cardiometabolic disease (diabetes) | Update to plan, setting out how commitments to improved diabetes care will delivered |  |
| [67] | Heart Conditions Delivery Plan | 06/01/2017 | NHS Wales | Wales | Action plan | Cardiometabolic disease | Updated delivery plan for prevention of heart disease, and care of people with heart conditions |  |
| [68] | The quality statement for heart conditions | 22/03/2021 | Welsh Government | Wales | Policy paper | Cardiometabolic disease | Replaces the Heart Conditions Delivery Plan. Sets out plans to improve short-term recovery and longer-term transformation of cardiac services. |  |
| [69] | Illnesses and long-term conditions | 2021 | Scottish government | Scotland | Policy paper | Cardiometabolic disease | Sets out overview and actions in relation to population health; we focus specifically on heart disease and diabetes |  |
| Policy, strategies, action plans related to COVID-19 | | | | | | | | |
| [70] | UK COVID-19 vaccines delivery plan - GOV.UK | 13/01/2021 | Department of Health and Social care | UK | Action plan | Public health | Sets out plan to deliver COVID-19 vaccines in the UK, including prioritisation and working with communities. |  |
| [71] | UK COVID-19 vaccine uptake plan | 13/02/2021 | Department of Health and Social care | UK | Action plan | Public health | Builds on delivery plan, sets out how government will increase vaccine uptake. |  |
| [72] | COVID-19 Mental Health and wellbeing recovery action plan | 27/03/21 | Department of Health and Social care | England | Action plan | Public health | Sets out measures to prevent, mitigate and respond to mental health impacts of the pandemic during 2021-22 |  |
| [73] | Adult social care: COVID-19 winter plan 2021 to 2022 | 03/11/21 | Department of health and social care | UK | Action plan | Public health | Set out support available for adult social care sector for winter 2021-22, and actions for relevant organisations. |  |
| Policy, strategies, action plans related to public health or other relevant health conditions | | | | | | | | |
| [74] | Leeds Health and Wellbeing Strategy 2016-2021 | 04/07/16 | Leeds Clinical Commissioning Group | Leeds (England) | Policy paper | Public health | Sets out strategy for improving health and wellbeing in Leeds, with a focus on reducing health inequalities |  |
| [75] | Health and Wellbeing 2026: Delivering Together | 26/10/16 | Department of health | Northern Ireland | Policy paper | Public health | Strategy for change of the health and social care system. |  |
| Policy, strategies, action plans related to inequalities, including health inequalities | | | | | | | | |
|  | Northern Ireland: Health Inequalities Annual Report   - Health Inequalities Annual Report 2018 (first year) [76] - Health Inequalities Annual Report 2019 [77] - Health Inequalities Annual Report 2020 [78] - Health Inequalities Annual Report 2021 [79] - Health Inequalities Annual Report 2022 [80] | Annual | Department of health | Northern Ireland | Corporate report | Health inequalities | Annual report on national and regional health inequalities |  |
| [81] | Mind the Gap: Building bridges to better health for all (Kent’s Health Inequalities Action Plan 2012/15) | 2012 | Kent County Council | Kent (England) | Action plan | Health inequalities | Sets out who is responsible, and priorities and actions for addressing health inequalities in the region. |  |
| [82] | PHE equality objectives: 2017 to 2020 | 21/02/2017 | PHE | England | Corporate report | Health inequalities | Sets out how PHE will comply with the public sector equality duty |  |
| [83] | Health Improvement | 2019 | Scottish government | Scotland | Policy paper | Health inequalities | Sets out policy on reducing health gap in Scotland, including policy actions and delivery plan on diet and healthy weight |  |
| [84] | Digital technology and health inequalities: a scoping review | 2020 | Public health Wales | Wales | Report | Health inequalities, digital health | Scoping review carried out for Public Health Wales, which looked at existing patterns of digital health inequalities, and evidence for mitigating these effects. |  |
| [85] | Disparities in the risk and outcomes of COVID-19 | 02/06/2020 | Public Health England | England | Report | Health inequalities | Review of data on the disparities in the risks and outcomes from COVID-19 |  |
|  | Commission on race and ethnic disparities   - Summary of responses to the call for evidence [86] - Report [87] - Sub-group priorities [88] | 2020-21 | Commission on race and ethnic disparities | UK | Documents linked to the commission | Health inequalities; ethnicity | Reviews inequality in the UK, focusing on areas including poverty, education, employment, health and the criminal justice system. |  |
| [89] | Our vision for the women's heath strategy for England | 23/12/2021 | Department of health and Social care | UK | Policy paper | Health inequalities; cardiometabolic disease | Strategy for improving women’s health, and access to, and experience of, health services. |  |
| [90] | Inclusive Britain: government response to the Commission on Race and Ethnic Disparities | 17/03/2022 | HM Government | UK | Policy paper | Health inequalities | Response to the Commission on Race and Ethnic Disparities |  |

**References:**

1 Department of Health. The power of information: Putting all of us in control of the health and care information we need. 2012;:119.https://www.gov.uk/government/publications/giving-people-control-of-the-health-and-care-information-they-need

2 Department of Health and Social Care. Digital strategy: Leading the culture change in health and care. 2012;:1–32.papers3://publication/uuid/29A946E2-0228-46C9-A8E4-96EC3A1DD114

3 WHO. Promoting sport and enhancing health in European Union countries: *WHO Reg Off Eur* 2011;:64.https://apps.who.int/iris/handle/10665/108595

4 Bengtsson M. How to plan and perform a qualitative study using content analysis. *NursingPlus Open* 2016;**2**:8–14. doi:10.1016/j.npls.2016.01.001

5 Braun V, Clarke V. Using thematic analysis in psychology. *Qual Res Psychol* 2006;**3**:77–101. doi:10.1191/1478088706QP063OA

6 Frith H, Gleeson K. Clothing and Embodiment: Men Managing Body Image and Appearance. *Psychol Men Masculinity* 2004;**5**:40–8. doi:10.1037/1524-9220.5.1.40

7 Department of Health. The Mandate A mandate from the Government to the NHS Commissioning Board: April 2013 to March 2015. Published Online First: 2013.https://www.gov.uk/government/publications/the-nhs-mandate (accessed 19 May 2022).

8 Department of Health. The Mandate A mandate from the Government to NHS England: April 2015 to March 2016. 2015.https://www.gov.uk/government/publications/nhs-mandate-2015-to-2016 (accessed 19 May 2022).

9 Department of Health. The Government’s mandate to NHS England for 2016-17. 2017.https://www.gov.uk/government/publications/nhs-mandate-2016-to-2017 (accessed 19 May 2022).

10 Department of Health. The Government’s revised mandate to NHS England for 2017-18. 2018.https://www.gov.uk/government/publications/nhs-mandate-2017-to-2018 (accessed 25 May 2022).

11 Department of Health and Social Care. The Government’s revised mandate to NHS England for 2018-19. 2019.https://www.gov.uk/government/publications/nhs-mandate-2018-to-2019 (accessed 25 May 2022).

12 Department of Health and Social Care. The Government’s revised 2019-20 Accountability Framework with NHS England and NHS Improvement. 2020.https://www.gov.uk/government/publications/nhs-accountability-framework-2019-to-2020 (accessed 25 May 2022).

13 Department of Health and Social Care. The Government’s revised 2020-2021 mandate to NHS England and NHS Improvement. 2021.https://www.gov.uk/government/publications/nhs-mandate-2020-to-2021 (accessed 25 May 2022).

14 Department of Health and Social Care. The Government’s revised 2021-22 mandate to NHS England and NHS Improvement. 2022.https://www.gov.uk/government/publications/nhs-mandate-2021-to-2022 (accessed 25 May 2022).

15 Department of Health and Social Care. The Government’s 2022-23 mandate to NHS England. 2022.https://www.gov.uk/government/publications/nhs-mandate-2022-to-2023 (accessed 25 May 2022).

16 Public Health England. Our priorities for 2013/14. 2013.https://www.gov.uk/government/publications/public-health-englands-priorities-for-2013-to-2014 (accessed 17 May 2022).

17 Public Health England. PHE remit letter: 2014 to 2015. 2014.https://www.gov.uk/government/publications/phe-remit-letter-2014-to-2015 (accessed 17 May 2022).

18 Public Health England. PHE remit letter: 2015 to 2016. 2015.https://www.gov.uk/government/publications/phe-remit-letter-2015-to-2016 (accessed 17 May 2022).

19 Public Health England. PHE remit letter: 2016 to 2017. 2016.https://www.gov.uk/government/publications/phe-remit-letter-2016-to-2017 (accessed 17 May 2022).

20 Public Health England. PHE remit letter: 2017 to 2018. 2017.https://www.gov.uk/government/publications/phe-remit-letter-2017-to-2018 (accessed 17 May 2022).

21 Public Health England. PHE priorities in health and care: 2018 to 2019. 2018.https://www.gov.uk/government/publications/phe-remit-letter-2018-to-2019 (accessed 17 May 2022).

22 Public Health England. PHE priorities in health and social care: 2019 to 2020. 2019.https://www.gov.uk/government/publications/phe-priorities-in-health-and-social-care-2019-to-2020 (accessed 17 May 2022).

23 Department of Health and Social Care. PHE priorities in health and social care: 2020 to 2021. 2020. https://www.gov.uk/government/publications/phe-priorities-in-health-and-social-care-2020-to-2021 (accessed 21 Apr 2022).

24 Department of Health. Delivering high quality, effective, compassionate care: Developing the right people with the right skills and the right values. A mandate from the Government to Health Education England: April 2013 to March 2015 May. 2013.https://www.gov.uk/government/publications/health-education-england-mandate (accessed 27 May 2022).

25 Department of Health. Delivering high quality, effective, compassionate care: Developing the right people with the right skills and the right values. A mandate from the Government to Health Education England: April 2014 to March 2015. 2014.https://www.gov.uk/government/publications/health-education-england-mandate-april-2014-to-march-2015 (accessed 27 May 2022).

26 Department of Health. Delivering high quality, effective, compassionate care: Developing the right people with the right skills and the right values. A mandate from the Government to Health Education England: April 2015 to March 2016. 2015.https://www.gov.uk/government/publications/health-education-england-mandate-april-2015-to-march-2016 (accessed 27 May 2022).

27 Department of Health. Delivering high quality, effective, compassionate care: Developing the right people with the right skills and the right values. A mandate from the Government to Health Education England: April 2016 to March 2017. 2016.https://www.gov.uk/government/publications/hee-mandate-2016-to-2017 (accessed 27 May 2022).

28 Department of Health and Social Care. The Department of Health and Social Care mandate to Health Education England April 2017 to March 2018. 2018.https://www.gov.uk/government/publications/health-education-england-mandate-2017-to-2018 (accessed 27 May 2022).

29 Department of Health and Social Care. Department of Health and Social Education England: April 2018 to Care mandate to Health March 2019. 2019.https://www.gov.uk/government/publications/health-education-england-mandate-2018-to-2019 (accessed 27 May 2022).

30 Department of Health & Social Care. The Department of Health and Social Care mandate to Health Education England: April 2019 to March 2020. 2019.https://www.gov.uk/government/publications/health-education-england-mandate-2019-to-2020 (accessed 27 May 2022).

31 Department of Health & Social Care. The Department of Health and Social Care mandate to Health Education England: April 2020 to March 2021. 2020.https://www.gov.uk/government/publications/health-education-england-mandate-2020-to-2021 (accessed 27 May 2022).

32 Department of Health & Social Care. The Department of Health and Social Care mandate to Health Education England: April 2021 to March 2022 - GOV.UK. 2021.https://www.gov.uk/government/publications/health-education-england-mandate-2021-to-2022/the-department-of-health-and-social-care-mandate-to-health-education-england-april-2021-to-march-2022 (accessed 27 May 2022).

33 NHS Scotland. NHS Scotland Chief Executive’s Annual Report 2012/13. 2013.https://www.gov.scot/publications/nhsscotland-chief-executives-annual-report-2012-13/documents/ (accessed 27 May 2022).

34 NHS Scotland. NHS Scotland Chief Executive’s Annual Report 2013/14. 2014.https://www.gov.scot/publications/nhsscotland-chief-executives-annual-report-2013-14/documents/ (accessed 27 May 2022).

35 NHS Scotland. NHS Scotland Chief Executive’s Annual Report 2014/15. 2015.https://www.gov.scot/publications/nhsscotland-chief-executives-annual-report-2014-15/documents/ (accessed 27 May 2022).

36 NHS Scotland. NHS Scotland Chief Executive’s Annual Report 2015/2016. 2016.https://www.gov.scot/publications/nhsscotland-chief-executives-annual-report-2015-16/

37 NHS Scotland. NHS Scotland chief executive’s annual report 2016/17. 2017.https://www.gov.scot/publications/nhsscotland-chief-executives-annual-report-2016-17/documents/ (accessed 27 May 2022).

38 NHS Scotland. NHS Scotland Chief Executive’s Annual Report 2017/18. 2018.https://www.gov.scot/publications/nhs-scotland-chief-executives-annual-report-2017-18/ (accessed 27 May 2022).

39 NHS England. NHS Five Year Forward View. 2014. https://www.england.nhs.uk/publication/nhs-five-year-forward-view/

40 Public Health Wales. Our Strategic Plan (Integrated Medium Term Plan) 2019-2022. Published Online First: 2018.https://phw.nhs.wales/about-us/our-priorities/long-term-strategy-documents/public-health-wales-strategic-plan-2019-22/

41 Cabinet Office, Department of Health and Social Care. Advancing our health: prevention in the 2020s. 2019.https://www.gov.uk/government/consultations/advancing-our-health-prevention-in-the-2020s (accessed 27 May 2022).

42 England PH. PHE Strategy 2020 to 2025. 2019.https://www.gov.uk/government/publications/phe-strategy-2020-to-2025 (accessed 21 Apr 2022).

43 Department of Health and Social Care. Integration and Innovation: working together to improve health and social care for all. 2021.https://www.gov.uk/government/publications/working-together-to-improve-health-and-social-care-for-all

44 Transforming the public health system: reforming the public health system for the challenges of our times - GOV.UK. https://www.gov.uk/government/publications/transforming-the-public-health-system/transforming-the-public-health-system-reforming-the-public-health-system-for-the-challenges-of-our-times (accessed 27 May 2022).

45 NHS Scotland. Recover, Restore, Renew. Chief Medical Officer Annual report 2020 to 2021. 2021.https://www.gov.scot/publications/cmo-annual-report-2020-21/ (accessed 27 May 2022).

46 Build Back Better: Our Plan for Health and Social Care - GOV.UK. https://www.gov.uk/government/publications/build-back-better-our-plan-for-health-and-social-care

47 Welsh Government. Digital Action Plan 2014 - 2017. 2014.

48 Department for Digital, Culture M and S. UK Digital Strategy - GOV.UK. Policy Pap. 2017.https://www.gov.uk/government/publications/uk-digital-strategy (accessed 21 Apr 2022).

49 Scottish Government. Realising Scotland’s full potential in a digital world: a digital strategy for Scotland. 2017. http://www.gov.scot/Resource/0051/00515583.pdf

50 Welsh Government. Improving people’s lives through digital technologies: Digital Inclusion Progress Report and Forward Look 2018. 2018;:13.https://gov.wales/sites/default/files/publications/2019-05/digital-inclusion-framework-report-and-forward-look.pdf

51 Department of Business, Energy, and Industrial Strategy; Ministry of Housing C and LG. West Midlands Local Industrial Strategy. 2019.https://www.gov.uk/government/publications/west-midlands-local-industrial-strategy (accessed 27 May 2022).

52 Welsh Government. Digital Strategy for Wales. 2021.https://gov.wales/digital-strategy-wales-html

53 TEC. TEC At a Glance 2019. Published Online First: 2020.https://tec.scot/sites/default/files/2021-02/TEC-At-a-Glance-2019.pdf (accessed 27 May 2022).

54 TEC. TEC At a Glance 2020/21. 2021.

55 Board NI. Personalised health and care 2020. https://www.gov.uk/government/publications/personalised-health-and-care-2020 (accessed 21 Apr 2022).

56 Welsh Government. Informed Health and Care: A Digital Health and Social Care Strategy for Wales. Published Online First: 2015.https://gov.wales/digital-health-and-social-care-strategy

57 Health and Social Care Board. eHealth and Care Strategy for Northern Ireland. Published Online First: 2016.https://www.health-ni.gov.uk/publications/ehealth-and-care-strategy

58 NHS England. Healthy Children: Transforming Child Health Information. Published Online First: 2016.https://www.england.nhs.uk/publication/healthy-children-transforming-child-health-information-november-2016/ (accessed 1 Apr 2022).

59 Digital-first public health - GOV.UK. https://www.gov.uk/government/publications/digital-first-public-health (accessed 26 May 2021).

60 Digital Health and Care Scotland. Scotland’s Digital Health and Care Strategy: enabling, connecting and empowering. 2018;:20.https://www.gov.scot/publications/scotlands-digital-health-care-strategy-enabling-connecting-empowering/ (accessed 18 May 2022).

61 NHS Lanarkshire. Digital Strategy 2019-2026. 2019;:1–40.https://www.nhslanarkshire.scot.nhs.uk/download/digital-strategy-2019-2026/

62 GOV.UK. Data saves lives: reshaping health and social care with data (draft). 2021.

63 Government S. Enabling, Connecting and Empowering: Care in the Digital Age. *Refreshed Digit Heal care Strateg* Published Online First: 2021.https://www.gov.scot/publications/scotlands-digital-health-care-strategy/ (accessed 18 May 2022).

64 Digital Health and Care Scotland. Digital Citizen Delivery Plan 2021/2022. 2021.https://tec.scot/sites/default/files/2021-06/Digital-Citizen-Delivery-Plan-final-21-22.pdf

65 Department of Health and Social Care. G7 patient access to health records: final report. 2021. https://www.gov.uk/government/publications/g7-health-track-digital-health-final-reports/g7-patient-access-to-health-records-final-report

66 NHS Wales, Welsh Government. Diabetes Delivery Plan for Wales 2016-2020. 2016;:22.https://gov.wales/diabetes-delivery-plan-2016-2020

67 Welsh Government. Heart Conditions Delivery Plan. 2017;:30.https://gov.wales/sites/default/files/publications/2019-01/heart-conditions-delivery-plan-january-2017.pdf

68 Welsh Government. The quality statement for heart conditions. 2021.https://gov.wales/quality-statement-heart-conditions

69 NHS Inform. Illnesses and Conditions. Webpage. 2019;:Mental Health.https://www.gov.scot/policies/illnesses-and-long-term-conditions/ (accessed 1 Apr 2022).

70 Department of Health and Social Care. UK COVID-19 vaccines delivery plan. 2021.https://www.gov.uk/government/publications/uk-covid-19-vaccines-delivery-plan (accessed 27 May 2022).

71 Department of Health and Social Care. UK COVID-19 vaccine uptake plan. 2021;:1–14.https://www.gov.uk/government/publications/covid-19-vaccination-uptake-plan/uk-covid-19-vaccine-uptake-plan

72 Department of Health and Social Care. COVID-19 Mental Health and Wellbeing Recovery Action Plan. 2021;:1–55.https://www.gov.uk/government/publications/covid-19-mental-health-and-wellbeing-recovery-action-plan

73 Department of Health and Social Care. Adult social care: COVID-19 winter plan 2021 to 2022. 2021. https://www.gov.uk/government/publications/adult-social-care-coronavirus-covid-19-winter-plan-2021-to-2022/adult-social-care-covid-19-winter-plan-2021-to-2022

74 Leeds Health and Wellbeing Board. Leeds Health and Wellbeing Strategy 2016-2021. Published Online First: 2016.http://inspiringchangeleeds.org/wp-content/uploads/2018/08/Health-and-Wellbeing-2016-2021-WEB.pdf

75 Department of Health (Northern Ireland). Health and Wellbeing 2026: Delivering Together. Published Online First: 2016.https://www.health-ni.gov.uk/sites/default/files/publications/health/health-and-wellbeing-2026-delivering-together.pdf

76 Health Inequalities – Annual Report 2018 | Department of Health. https://www.health-ni.gov.uk/news/health-inequalities-annual-report-2018 (accessed 26 May 2022).

77 Health Inequalities Annual Report 2019 | Department of Health. https://www.health-ni.gov.uk/news/health-inequalities-annual-report-2019 (accessed 26 May 2022).

78 Department of Health (Northern Ireland). Health Inequalities Annual Report 2020. 2020.https://www.health-ni.gov.uk/news/health-inequalities-annual-report-2020 (accessed 1 Apr 2022).

79 Department of Health (Northern Ireland). Health Inequalities Annual Report 2021. 2021.https://www.health-ni.gov.uk/news/health-inequalities-annual-report-2021 (accessed 1 Apr 2022).

80 Health inequalities annual report 2022 | Department of Health. https://www.health-ni.gov.uk/news/health-inequalities-annual-report-2022 (accessed 26 May 2022).

81 Kent County Council. Mind the Gap: Building bridges to better health for all 2012/15. ;:1–62.https://www.kent.gov.uk/__data/assets/pdf_file/0008/14777/Mind-the-Gap-Building-bridges-to-better-health-for-all.pdf

82 Health England P. PHE equality objectives for 2017 to 2020. Published Online First: 2017.https://www.gov.uk/government/publications/phe-equality-objectives-2017-to-2020 (accessed 19 May 2022).

83 Health and Social Care SG. Health improvement. 2019.https://www.gov.scot/policies/health-improvement/ (accessed 1 Apr 2022).

84 Honeyman M, Maguire D, Evans H, *et al.* Digital technology and health inequalities: a scoping review. 2020. www.nationalarchives.gov.uk/doc/open-government-licence/version/3/ (accessed 20 Apr 2021).

85 Public Health England. Disparities in the risk and outcomes of COVID-19. 2020.https://www.gov.uk/government/publications/covid-19-review-of-disparities-in-risks-and-outcomes (accessed 26 May 2022).

86 Commission on Race and Ethnic Disparities: Summary of responses to the call for evidence. https://www.gov.uk/government/consultations/ethnic-disparities-and-inequality-in-the-uk-call-for-evidence/public-feedback/summary-of-responses-to-the-call-for-evidence (accessed 26 May 2022).

87 The report of the Commission on Race and Ethnic Disparities - GOV.UK. https://www.gov.uk/government/publications/the-report-of-the-commission-on-race-and-ethnic-disparities (accessed 26 May 2022).

88 Commission on Race and Ethnic Disparities. Commission on Race and Ethnic Disparities: sub-group priorities. https://www.gov.uk/government/publications/commission-on-race-and-ethnic-disparities-sub-group-priorities (accessed 27 May 2022).

89 Department of Health & Social Care. Our Vision for the Women’s Health Strategy for England. 2021. https://www.gov.uk/government/publications/our-vision-for-the-womens-health-strategy-for-england

90 HM Government. Inclusive Britain: government response to the Commission on Race and Ethnic Disparities. https://www.gov.uk/government/publications/inclusive-britain-action-plan-government-response-to-the-commission-on-race-and-ethnic-disparities (accessed 27 May 2022).
